# Temporal tuning in the bat auditory cortex is sharper when studied with natural echolocation sequences

**DOI:** 10.1038/srep29102

**Published:** 2016-06-30

**Authors:** M. Jerome Beetz, Julio C. Hechavarría, Manfred Kössl

**Affiliations:** 1 Institut für Zellbiologie und Neurowissenschaft, Goethe-Universität, 60438, Frankfurt/M., Germany

## Abstract

Precise temporal coding is necessary for proper acoustic analysis. However, at cortical level, forward suppression appears to limit the ability of neurons to extract temporal information from natural sound sequences. Here we studied how temporal processing can be maintained in the bats’ cortex in the presence of suppression evoked by natural echolocation streams that are relevant to the bats’ behavior. We show that cortical neurons tuned to target-distance actually profit from forward suppression induced by natural echolocation sequences. These neurons can more precisely extract target distance information when they are stimulated with natural echolocation sequences than during stimulation with isolated call-echo pairs. We conclude that forward suppression does for time domain tuning what lateral inhibition does for selectivity forms such as auditory frequency tuning and visual orientation tuning. When talking about cortical processing, suppression should be seen as a mechanistic tool rather than a limiting element.

Precise temporal processing is crucial in the auditory system. It is important for distinguishing phonemes and words in human language^1^. Auditory deficits like “word deafness” are associated with decreased temporal precision at the level of the primary auditory cortex^2^,^3^.

Physiologically, cortical neurons show strong forward suppression^4^,^5^ resulting in decreased ability of temporal tracking^6^. The present study tries to investigate how the echolocation system of bats, and more precisely how cortical neurons tuned to specific target-distances cope with fast auditory stimuli that should induce strong suppression effects.

Bats are animal models suited very well for investigating temporal processing in the auditory system. During echolocation, they broadcast sequences of calls at high repetition rates (Fig. 1a). For example, in the bat species *Carollia perspicillata,* the minimal intercall time intervals are around 20 ms^7^. The bats calculate the distance of objects with the help of the delay of returning echoes and neurons that are tuned to echo-delay are a characteristic feature of auditory cortex of bats^8^. Inactivation of such neurons leads to deficits in echo-delay perception^9^. Although target-distance neurons were discovered in the 1970’s, studies on delay tuning so far have focussed mainly on describing neuronal receptive fields by using simulated pairs of biosonar call and echo. Little is known about the responses of delay-tuned neurons when the bat faces natural echolocation streams.

The main goal of the present paper was to describe how the temporal arrangement of information in the biosonar sequences affects the response of cortical delay-tuned neurons. To achieve this goal, we compared the response to natural echolocation sequences with an “expected response”, estimated by presenting the single call-echo elements that composed the natural echolocation sequences in a random order and at long (400 ms) inter-call time-intervals. Our results show that strong suppression dominates the response of delay-tuned neurons to natural echolocation sequences. However, the suppression is non-uniform, because responses to specific call-echo elements, whose echo delays represent the neurons’ “best” echo delay, are less suppressed than responses to neighbouring call-echo elements. In addition, delay-specific facilitation adds to a sharpening of the neuronal response to the natural echolocation sequence.

## Results

### Natural echolocation sequence

**Figure 1:**
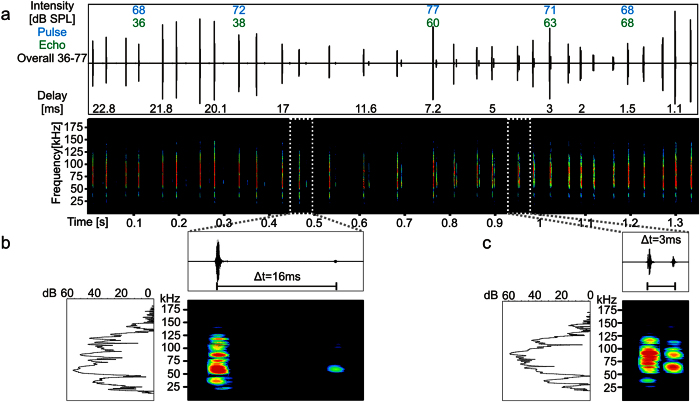
Natural echolocation sequence used as stimulus for electrophysiology recordings. **(a)** Oscillogram and spectrogram of the echolocation sequence. The sound pressure levels at the maximal level ranged between 36–77 dB SPL. The intensities of selected call-echo elements are indicated in blue and green for call and echo, respectively. Echo delays ranged from 22.8 to 1.1 ms. The temporal delays between call and echo are indicated in the lower part of the oscillogram for selected call-echo elements. **(b**,**c)** Magnified oscillogram, spectrogram and power spectrum from two example call-echo elements with echo delays of 16 and 3 ms (1024 samples; Hanning window). See also Supplementary Table 1.

A representative echolocation sequence for an approach flight was recorded from a bat that was swung in a pendulum (Fig. 1a, method after^10^) towards an acrylic glass wall. During the swing the animal echolocated and the broadcasted calls and their echoes, arising from call reflections from the wall, were recorded with an ultrasonic microphone located on top of the animal at 4 cm distance to the ears. The echolocation sequence used as natural stimulus, consisted of 31 call-echo elements. Echo delays decreased from 22.8 ms to 1.1 ms which corresponds to a decrease of object distance between 388 to 19 cm. Magnified call spectrograms (Fig. 1b,c) show that the main energy of the calls was between 50 and 100 kHz (for details see Supplementary Table 1).

### Neuronal response of echo delay-tuned neurons to natural echolocation sequence

**Figure 2:**
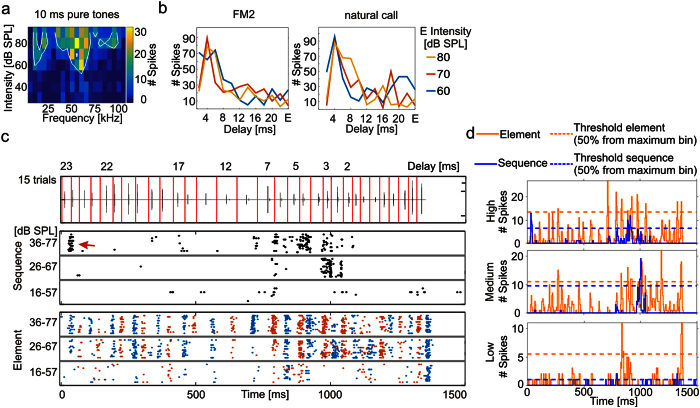
Responses of one example unit to temporally isolated call-echo elements and to the natural echolocation sequence. **(a)** Frequency tuning curve calculated from responses to different intensity-frequency combinations of a pure tone. The tuning curve demonstrates that the unit is sensitive to high frequencies. (**b)** Delay tuning curves calculated from neuronal responses to artificial frequency modulations that simulated the second harmonic of the bats’ calls (left) or to natural call-echo pairs (right). Different combinations of call-echo delays (2–22 ms in steps of 2 ms) and echo sound levels (80, 70 and 60 dB SPL) were presented to the animal. The interstimulus time interval was set to 400 ms. The delay tuning curves show that the unit responded strongly to short delays at around 4 ms. Responses to the echo alone [E] without preceding call is given in the last vertical bins of the data field. (**c)** Top: Oscillogram of the echolocation sequence. Vertical red lines indicate time borders between different call-echo elements that were used for segmenting the sequence. Bottom: Raster plots calculated from the response to the echolocation sequence (black dots) and to the elements (colored dots), where the call-echo elements were randomly presented with a 400 ms interstimulus time interval. To visualize which spikes were elicited in responses to which call-echo element, the responses to consecutive call-echo elements are indicated through alternating colors. Red arrow points to a characteristic initial response. Horizontal gray lines separate raster plots obtained at three sound levels. (**d)** Post-stimulus time histograms (PSTHs; binsize = 5 ms) for the three sound levels and for the sequence (blue) and element (orange) situation. Note that the response to the sequence is suppressed resulting in temporally sharper responses than those in the element situation. Horizontal dashed orange and blue lines indicate thresholds (50% from maximum bin). See also Supplementary Fig. 1.

Electrophysiological recordings were performed with either commercially available tungsten microwire arrays with 16 channels (Tucker Davis Technologies, USA) or with custom-built 4–6 channels glass electrode arrays. The arrays enabled simultaneous recordings from several locations of *C. perspicillata*’s high frequency area of the auditory cortex, where target-distance neurons are located^11^. In total, 149 cortical units (38 units with tungsten microwire arrays and 111 units with glass electrode arrays) were recorded from 10 anesthetized animals from both brain hemispheres (5 females, 5 males). The frequency tuning curves were characteristic for delay-tuned neurons^11^, with high sensitivity at around 60 kHz (Fig. 2a). Additionally, the units responded selectively to specific echo delays when they were stimulated with pairs of downward frequency modulated sounds (mimicking the second harmonic of *C perspicillata*’s call) and respond weakly to single downward frequency modulated sounds (Fig. 2b). Delay tuning curves calculated with natural call-echo elements resulted in comparable delay tuning.

To investigate the relevance of the temporal context of the stimuli, we split the echolocation sequence into 31 call-echo elements (Fig. 2c; red vertical lines in the oscillogram indicate time borders) and played them in a randomized fashion with 400 ms interstimulus time intervals. This paradigm is addressed as the “element situation” in contrast to the “sequence situation” in which the echolocation sequence was played in its natural form. Natural stimuli were played at three different sound levels thus covering sound pressure levels that were classified as: (i) high: 36–77 dB SPL, (ii) medium: 26–67 dB SPL; and (iii) low: 16–57 dB SPL (for details see Supplementary Table 1). The spike-times obtained in response to each element in the “element situation” were temporally aligned to the time point of that element in the echolocation sequence (Fig. 2c, bottom: colored raster plots) to create an expected response.

In the “sequence situation” we observed that the firing rate of the units was lower than that observed in response to the “element situation” (compare black (middle) versus colored (bottom) raster plots in Fig. 2c). Some units showed an initial response to the first sequence elements after about 40 ms before they were strongly suppressed (Fig. 2c; red arrow in raster plot). Such initial responses occurred in 68.5%, 69.1% and 57.1% in the recordings obtained at high, medium and low sound levels, respectively. The initial response was not crucial for inducing suppression. In the example given in Fig. 2c, for the medium sound level of 26–67 dB SPL, strong suppression was evident despite a lack of initial response.

During the “sequence situation”, the overall suppressive effect was strong, however, the units were not suppressed for the entire duration of the sequence. Instead, at some time points, the units recovered from suppression and responded more selectively to specific call-echo elements than in the “element situation”. The suppression and sharpening effect can be viewed directly by comparing the post-stimulus time histograms (PSTHs; binsize = 5 ms) from the example unit (Fig. 2d). Note that in this particular unit, a temporally sharp response to certain portions of the echolocation sequence was also evident in response to other natural and artificial echolocation sequences (Supplementary Fig. 1).

### Quantification of the response suppression

**Figure 3:**
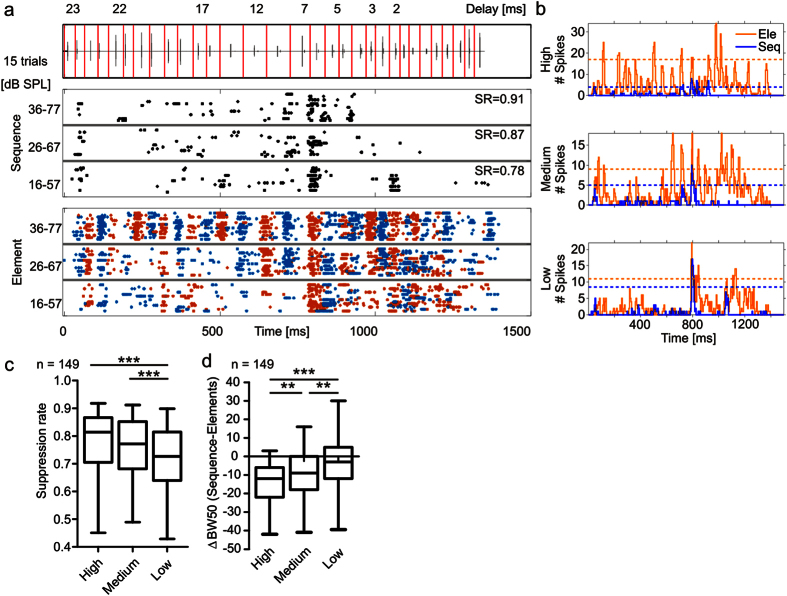
Quantification of suppression induced by behaviorally relevant intercall time intervals. **(a)** Raster plots of a unit showing sound level dependent suppression. Suppression rate (SR) for each sound level is indicated. (**b)** PSTHs of the unit for each sound level and each stimulus situation (element = orange; sequence = blue). Organization as in Fig. 2. (**c**) Boxplots (whiskers represent 5–95% percentile) showing the suppression rates of all multi-units. **(d)** Boxplots (whiskers represent 5–95% percentile) showing the bandwidth differences between sequence and element situation for each sound level. Friedman and Dunn’s multiple comparison post hoc test in (**c**,**d)** (**p < 0.01; *** p = < 10^−5^). See also Supplementary Fig. 2.

For quantifying the strength of suppression, we calculated a suppression rate. The suppression rate represents the ratio between the total number of spikes fired in the “sequence situation” (Fig. 3a; black raster plot and Fig. 3b blue PSTHs) and in the “element situation” (Fig. 3a; colored raster plot and Fig. 3b orange PSTHs) and subtracted the obtained ratio from 1. Thus, a suppression rate of 0.91 (Fig. 3a; high sound level) indicates that 91% less spikes occurred in the “sequence” than in the “element situation”. Despite of having variable suppression rates, all 149 units studied were suppressed in the “sequence situation” when compared to the “element situation”. For example, pooled suppression rates were well above 0, which is the value that would indicate no suppression (Fig 3c.) The suppression rates decreased significantly with sound level (Fig. 3c, Friedman and Dunn’s multiple comparison post hoc test; ***p < 10^−5^; n = 149).

For quantifying the impact of the suppression on the neuronal tuning, we calculated the 50% bandwidth from the PSTHs. For each unit, sound level and stimulus situation we used a different threshold that was set to the 50% from the bin that contained the maximum number of spikes in the corresponding situation. We did not use the same threshold for the “element” and “sequence situation” because in that case, a decreased bandwidth could result from a suppression that is equally strong over time, and which will not represent a sharper delay tuning (Figs 2d and 3b; dashed horizontal lines in the PSTHs). The number of bins surpassing the threshold were compared in the “element” and “sequence situation”. As expected, the units were more sharply tuned in the “sequence” than in the “element situation”, as indicated by a lower number of bins surpassing the threshold in the “sequence situation” (Fig. 3d). The sharpening effect decreased with decreasing sound level (Friedman and Dunn’s multiple comparison post hoc test; **p < 0.01; ***p < 10^−5^; n = 149). For the highest sound level, a linear correlation between the suppression and the sharpening effect was observed (Spearman r = −0.32; ***p < 10^−5^; n = 149; Supplementary Fig. 2).

### Suppression improves the topographic organization of echo-delays

**Figure 4:**
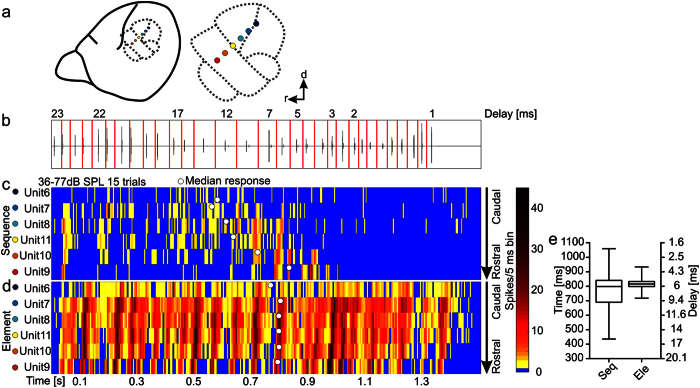
Suppression results in strong topographic organization of echo-delays along the rostro-caudal axis of the brain. **(a)** Schematic lateral view on *C. perspicillata*’s brain and magnified auditory cortical areas (dashed lines). Colored spots denote electrode positions from a single experiment. The linear electrode array was positioned along the rostro-caudal axis of the high frequency area and high frequency tuned neurons of the primary auditory cortex. d = dorsal, r = rostral. **(b)** Oscillogram of the echolocation sequence. Vertical red lines indicate borders between different call-echo elements that were used for segmenting the sequence. Echo delays ranged from 22.8 to 1.1 ms. **(c,d**) Color-maps with a binsize of 5 ms show the activity pattern of six multi-units in response to the “sequence” and “element situation” at the highest sound level. Each row represents the activity pattern from one unit in response to 15 averages. The units were recorded simultaneously from the auditory cortex and their positions follow the chronotopy along the rostro-caudal axis of the cortex (**a**). Note that the suppression leads to sharper delay tuning and to a more distinct topographic organisation along the rostro-caudal axis in the “sequence situation” **(c)**. In the element situation, the chronotopy is hardly detectable (**d)**. White dots in the color-maps represent time point of the median response for each unit. (**e)** Boxplots (whiskers represent minimum and maximum values) showing the distribution of median responses in 149 units. Note that more delays are covered in the “sequence” than in the “element situation”. Thus at the neuronal population more delays are represented in the “sequence situation”.

Cortical delay-tuned neurons are topographically organized in the rostro-caudal direction forming a chronotopic map^11^ in which shorter delays are represented rostrally and longer delays more caudally. Simultaneous recordings from six units along the rostro-caudal axis (Fig. 4a) allowed us to observe that the representation of echo delays along the cortical surface is affected by suppression. For example, the chronotopy becomes more distinct when tuning is studied in the “sequence” than in the “element situation” (compare Fig. 4c for “sequence situation” and Fig. 4d for “element situation”). Note that in Fig. 4, the discharge rate from each unit in 5 ms bins was transferred into a color-map, and the units were aligned along the rostro-caudal axis following the electrodes’ positions.

For quantifying the neuronal tuning in response to each stimulus situation at the population level we used the time points of all spikes in response to the whole sequence to calculate the median time of the overall response (“median response”; Fig. 4c,d; white dots). We did not use the best response as it has been done in former studies because with the best response only the time point of the maximum response is considered. In contrast the median response is calculated by considering all spikes and their time points of occurrence. When comparing the median responses from each unit for the “sequence” and “element situation”, it becomes clear that the median response values cover a larger delay range in the “sequence” than in the “element situation” (Fig. 4e). Thus cortical suppression widens the delay range that the chronotopic cortex is able to respond to. Additionally, in almost two third of the units (96 out of 149), the median response occurred earlier in the “sequence” than in the “element situation” (Wilcoxon signed rank test; p =  < 10^−5^, n = 149).

### Non-uniform suppression and facilitation shape cortical tuning

**Figure 5:**
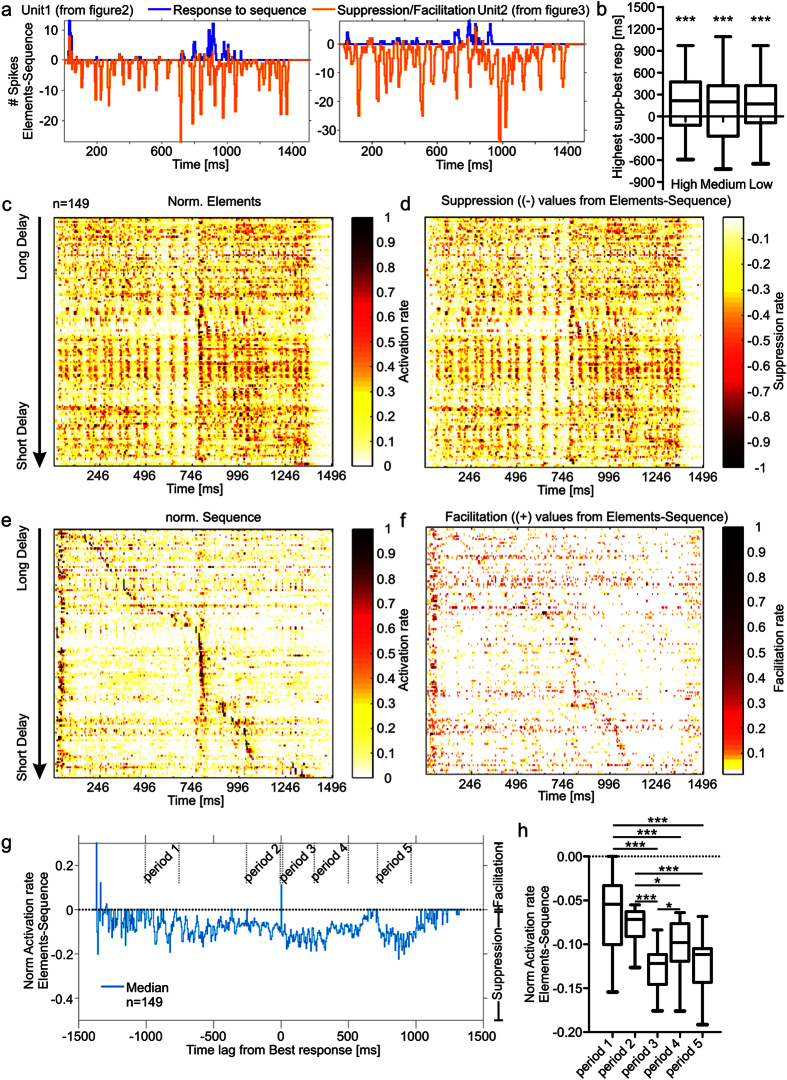
Interaction between non-uniform suppression and facilitation shapes the response to the natural echolocation sequence. **(a)** Response PSTH (blue) from the sequence situation and suppression/facilitation curve (orange) calculated by subtracting the element PSTH from the sequence PSTH, of two units stimulated at the highest sound level. Strong suppression occurs close to strong responses. (**b)** Temporal relationship between best response and strongest suppression for all studied sound levels. Strongest suppression occurs predominantly a few hundred ms after the best response. **(c)** Color-map of normalized response from each unit (organized with decreasing best delays along the y-axis) in response to the “element situation”. (**d)** Color-map of normalized amount of suppression from each unit. (**e)** Color-map of normalized response from each unit in response to the “sequence situation”. Note that areas of strongest response are much better defined than in (**c)**. (**f)** Color-map of normalized amount of facilitation from each unit. Note the facilitation pattern resembles the activation pattern in the “sequence situation”. **(g)** Median activation rate curve calculated from temporally aligned contrast PSTHs of each unit. Alignment was based on each neuron’s “best temporal bin” and thus best responses correspond to time point 0. The time periods used for statistical comparison are indicated. Each period covers 250 ms. **(h)** Statistical comparison of the median activation rates of the corresponding areas from (**g)**. Activation rates are more negative (higher suppression) in the three areas following the best response (period 4, 5 and 6) than in the areas preceding the best response (period 1, 2, 3). Note that (**c–h)** are exclusively presenting data obtained at the highest sound level. Sign test in (**b)**. Kruskal-Wallis and Dunn’s multiple comparison post hoc test in (**h)** (***p < 10^−5^). See also Supplementary Fig. 3.

For assessing the time course of suppression and facilitation we calculated suppression/facilitation curves by subtracting the PSTHs in the “element” from the PSTHs in the “sequence situation” in a bin-wise manner. Usually, strong suppression was in close temporal vicinity to strong responses (Fig. 5a and supplementary Fig. 3a). At the population level, maximal suppression occurred a few hundred milliseconds after the best response (median = 215 ms, 200 ms, 170 ms for high, medium and low sound levels, respectively; Fig. 5b; Sign test; ***p <10^−5^; n = 149, that indicates a distribution of values whose median deviates from zero).

The best delay of each unit was determined from its response to the “sequence situation” presented at the highest sound level. Best delay was calculated by relating the timing of the maximum response (that is, the bin with the highest spike count) to the call-echo delay occurring right before that time-point (see also Supplementary Fig. 3 for data on medium sound levels). Calculating “best delays” allowed us to relate the neuronal response and strength of suppression to the temporal tuning properties of the neurons.

To visualize the response patterns of all 149 units, normalized neuronal responses (normalized to the maximum spike count observed at the time bin of maximum responses) were ordered in decreasing order according to their best delay so that each row of Fig. 5c–f represents activity from one unit. This figure reveals that the pattern of overall excitation in the “element situation” (Fig. 5c) was matched by a similar pattern of broad suppression (Fig. 5d). Due to the effects of suppression, in the “sequence situation”, the temporal tuning was considerably sharper than in the “element situation” (compare Fig. 5e,c). There was an overrepresentation of units responding best to a specific call-echo element representing an echo delay of 7.2 ms (call # 16 in Supplementary Table 1, 22.1%, 33/149 units). Call # 16 had a sound level of 77 dB SPL and its echo, which was delayed by 7.2 ms from the start of the call, had a sound level of 60 dB SPL. Different parameters like sound level, frequency, delay, and stimulus history could be responsible for the observed response overrepresentation to this particular call-echo element.

In addition to strong, but relatively unspecific suppression, weak facilitation was found right at the best delay of the units (Fig. 5f). The corresponding population pattern matches that of the responses in the “sequence situation”. This result emphasizes that the response in the “sequence situation” is shaped through an interaction between widely spread suppression that is strongest after the best response, and facilitation occurring predominantly in the period of the best response.

To better quantify the timing of suppression/facilitation, we pooled the data from the 149 studied units by temporally aligning the normalized suppression/facilitation curves relative to each unit’s best response in the “sequence situation” (at the highest level, Supplementary Fig. 3 for data on medium intensity levels). The pooled suppression/facilitation curve shows a clear peak at 0 ms (Fig. 5g). This peak represents the facilitation occurring exactly at the time of the units’ best response to the sequence. Strong suppressive periods, indicated by negative values, were detected following the best response until 570 ms and between 715–1000 ms (Fig. 5g). For a statistical analysis, activation rates (calculated as the difference between the response to the sequence and that to the element situations) from three periods (period 3, 4, 5) following the best response were compared with values from two periods preceding the best response (period 1, 2). Each analysed period spanned a time of 250 ms. The activation rates of period 3, 4 and 5 were significantly smaller than the activation rates of period 1 and 2 (Fig. 5h; Kruskal-Wallis and Dunn’s multiple comparison post hoc test; ***p < 10^−5^; n = 149). In other words, this data indicates that the suppression happening after the best response is stronger than that happening before the best response. Note that for medium sound levels, comparable calculations revealed a large suppressive period that also was found after the best response, at between 1–600 ms but at this sound level the facilitation was sparser and temporally highly distributed which resulted in no clear peak at time point 0 (Supplementary Fig. 3).

### Impact of stimulus history on neuronal response

The fact that strong suppression usually follows the best response could be an indicator for forward suppression whose strength increases during the stimulation. To clarify the impact of preceding call-echo elements (stimulus history) on the response to consecutive elements, we temporally reversed the echolocation sequence but kept the individual call-echo elements in their original form, so that the call still precedes the echo and both are downward frequency modulated. In other words, the reversed echolocation sequence contains call-echo elements with increasing echo delays over time.

**Figure 6:**
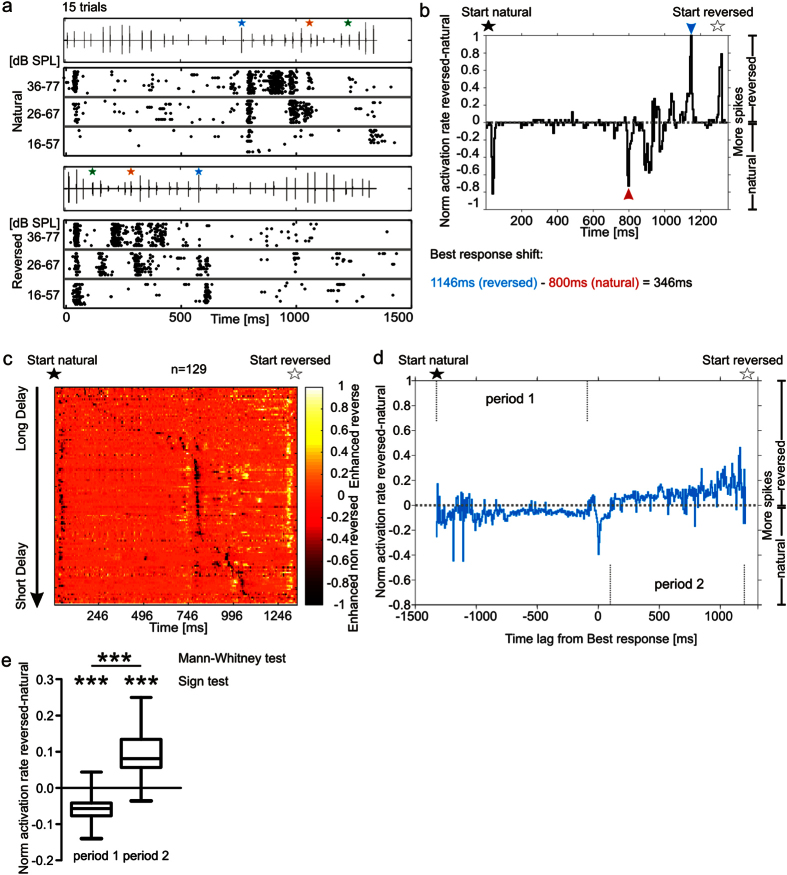
Stimulation with a temporally reversed echolocation sequence indicates a role of forward suppression shaping the time course of cortical responses. (**a**) Stimulus oscillogram and raster plots of a unit stimulated in the ”natural situation” (top) and “reversed situation” (bottom). Colored stars signal to specific call-echo elements leading to weaker responses in the “natural” than in the “reversed situation”. (**b**) Contrast response curve of the same unit. The normalized PSTH in the “natural situation” was subtracted from the temporally-mirrored normalized PSTH of the “reversed situation”. Positive values indicate more spikes while negative ones less spikes in the “reversed” than in the “natural situation”. Stars indicate the starting point of the corresponding stimulus (black and white for ”natural” and “reversed situation”, respectively). Blue and red arrowheads indicate the time point of best response in the “reversed” and “natural situation”, respectively. Note that the best response temporally shifts by 346 ms between the two situations. (**c**) Color-map of contrast response curves from each unit (organized with decreasing best delays along the y-axis). (**d**) Median contrast response curve calculated from temporally aligned contrast response curves of each unit. Bins without changes (values = 0) were excluded. Response curves were aligned so that the best responses in the “natural situation” correspond to time point 0. Note that negative values are dominant before and positive values after the zero point. (**e**) Values from period 1 and period 2 in (**d**) have medians that are statistically different from 0 (Sign test; ***p < 10^−5^, n = 129) and are also different from each other (Mann-Whitney test; ***p < 10^−5^; n = 129). See also Supplementary Fig. 4.

Neuronal activity in response to the natural and to the reversed echolocation sequence (“reversed situation”) were recorded. From the example unit in Fig. 6a it is clear that the unit shifted its response towards shorter delays in the “reversed situation” (compare the time ranges of strong excitations for each situation with the help of the colored stars marking specific calls in Fig. 6a). Additionally, more spikes were elicited in the first 500 ms of the “reversed situation” (lower panel in Fig. 6a) than in the last 500 ms of the “natural situation” (upper panel in Fig. 6a). Note that these two temporal windows covered the same call-echo elements in the sequences used as stimuli.

To visualize response pattern differences between the “reversed” and “natural situation” we calculated contrast response curves by subtracting the normalized activity in the “natural” from the “reversed situation”. For this subtraction, the PSTH in the “reversed situation” was temporally mirrored which enabled us to temporally align them to the PSTHs obtained from the “natural situation”. To exemplify this procedure, one contrast response curve, obtained at the highest sound level, is plotted in Fig. 6b. Positive and negative values indicate more spikes in the “reversed” and in the “natural situation”, respectively. For the population data obtained at high sound levels, contrast response curves were calculated for each unit (n = 129) and plotted into a color-map (Fig. 6c; see also Supplementary Fig. 4 where exclusively negative (a) or positive (b) values were plotted). In this figure, like in Fig. 5, the units are organized according to decreasing best delays in response to the “natural situation”. A median contrast response curve calculated from temporally aligned contrast curves of each unit, comparable to Fig. 5g, is plotted in Fig. 6d. Only bin values differing in the “natural” and “reversed situation” were considered. We observed that at long delays, representing the beginning of the “natural situation” (Fig. 6c,d; beginning from black stars), more spikes were elicited in the “natural” than in the “reversed situation” (Fig. 6e; Sign test for period 1; ***p < 10^−5^; n = 129). Consistent with the results presented in the previous section, this result suggests the presence of a relatively weak suppression at long delays in the “natural situation” that might arise from a missing stimulus history. In the “reversed situation”, a longer stimulus history and therefore a stronger suppression was observed at long delays.

Some units temporally shifted their best response when comparing the response to the “natural” and “reversed situation” (Fig. 6b) but considering the pooled data, the timing of best responses did not shift significantly between the two situations studied (Sign test p = 0.07; n = 129; median: 805 ms and 765 ms for “natural” and “reversed situation”, respectively; Supplementary Fig. 4c). Following the best response in the “natural situation”, the neuronal activity was significantly stronger in the “reversed situation” (Fig. 6e; Sign test for period 2; ***p < 10^−5^; n = 129). The latter could be the result of a stronger suppression operating in the “natural situation” and a relatively weaker suppression occurring in the “reversed situation” (Fig. 6c,d). Based on the different response patterns to the “reversed” and “natural situation”, we conclude that the stimulus history, indicated by the different echo delay distribution over time, has strong influence on how the neurons respond to the echolocation sequence. In addition, the fact that suppression increases over the time course of stimulation in the “natural” and “reversed situation”, suggests that echolocation sequence processing is strongly influenced by a forward suppression.

## Discussion

Temporal processing in the primary auditory cortex is crucial for proper auditory perception including speech^12^,^13^. Language consists of highly repetitive elements with short time intervals in between. Repetitive auditory stimuli usually lead to cortical suppression and the temporal precision of auditory responses degrades from the periphery towards the cortex^6^,^14^. How temporal processing quality can be maintained despite cortical suppression remains elusive. In the present study we wanted to clarify whether cortical suppression is also present in echolocating bats that are specialized for precise temporal coding. Our results show that cortical units of bats are strongly suppressed when they are stimulated with natural echolocation sequences. The sequences had a behaviorally relevant temporal context and order of call-echo elements (Figs 2, 3, 4). Instead of losing the sensitivity to specific call-echo elements due to the suppression, the units were more sharply tuned. The sharper tuning was based on a combination of weak temporally focussed facilitation, and widely spread suppression that is stronger at the time periods that follow the facilitation (Figs 5 and 6).

In the “element situation” the tuning seems to be unusually broad (Figs 2 and 3) when comparing to previous studies that used artificial call-echo elements^11^,^15^,^16^. Level relations between call and echo could influence the response strength. Delay tuning curves are often tilted such that with increasing echo level the tuning shifts towards shorter delays^11^,^15^,^17^,^18^. In the natural sequences, each call-echo element has a different level combination thus making it difficult to compare responses in this situation to those observed in more classic delay tuning curves (Fig. 2 and refs 8,19 for review). A second reason for having a broader tuning in the “element situation” could arise from spectral differences in the call-echo composition. The calls of C*. perspicillata* are spectrally similar to each other (Fig. 1b,c). However, based on the relatively broad frequency tuning of delay-tuned neurons it is conceivable that even small spectral differences in the call-echo composition can affect neuronal tuning (Fig. 1a)^11^.

Only a few studies exist trying to explain how temporal precision can be maintained under suppression^20^,^21^. There are two coding strategies that have been described concerning processing of highly repetitive stimuli. Either the neuronal response directly reflects the temporal structure of the sound stimuli (temporal code) or the neurons show sustained responses as soon as a specific stimulus repetition frequency is reached (rate code)^21^,^22^,^23^. According to our results from the “element situation”, the delay-tuned neurons might have the potential of temporally tracking the call-echo elements that compose a given echolocation sequence. However, in response to the “sequence situation”, the neurons do not temporally follow the echolocation calls but rather respond more selectively to certain portions of the sequences containing specific echo delays. Based on our results it remains unclear, how cortical neurons overcome the suppression in the “sequence situation” and respond in a highly selective manner to specific call-echo pairs ? 

Suppression was evident from the beginning of the stimulation stream, regardless of whether the first call-echo pairs did evoke spike activity or not. This is consistent with extracellular recordings from delay-tuned neurons of the cortex of *P. parnellii*^24^. Besides, *in vivo* patch clamp recordings from the auditory cortex of rats have shown that even subthreshold responses are followed by long lasting inhibition that causes a decrease in membrane potential^25^. Our results show that suppression builds up over the time course of stimulation (Fig. 6) until reaching its maximum usually a few hundreds of milliseconds after the best response which is consisting with the idea of inhibitory effects that accumulate over time (Fig. 5). Inhibitory effects in auditory and visual cortices have been shown to be strongest close to the best stimulus parameters^26^,^27^,^28^,^29^.

Note that in the present study the animals were anaesthetized with a mixture of xylazine and ketamine. The impact of anaesthesia on the cortical suppression described in the present study remains speculative.

The interaction between cortical inhibition and excitation is crucial for proper tuning in auditory, somatosensory and visual centres^16^,^29^,^30^,^31^,^32^,^33^,^34^,^35^,^36^,^37^. In principle two phenomena have been described to explain how inhibitory inputs can sharpen receptive fields. (i) Inhibitory inputs temporally follow excitatory ones, thus limiting the time window of excitation. (ii) Inhibitory neurons are more broadly tuned than excitatory ones, resulting in sharpening effects based on lateral inhibition^27^,^28^,^38^,^39^. Inhibition is not only crucial for shaping receptive fields but also for forward suppression. Cortical forward suppression has a short lasting component (50–100 ms) reflecting GABA-ergic intracortical inhibition^4^ and a long lasting component (more than 100 ms) induced at presynaptic sites of thalamocortical neurons^5^. Stimulus combination sensitivity as it is evident in responses to specific call-echo elemtns depends strongly on an interaction between excitation and inhibition^40^. Cortical delay-tuned neurons receive strong GABAergic input, although this does not seem to be involved in creating delay tuning per se^35^. Whether cortical GABAergic circuitry or subcortical inhibitory interactions are crucial for the suppression reported in the present study remains unsolved. It is possible, that cortical suppression elicits an “iceberg effect” via decreasing the excitability of neurons as it has already been proposed in shaping frequency tuning curves^27^,^38^ and in other sensory cortices^41^. Only stimuli evoking strong responses can overcome suppression and elicit spikes in suppressed neurons. Therefore, non-uniform or nonlinear suppression together with facilitatory effects could increase tuning sharpness (Fig. 5).

In bats, enhanced sharpness of duration and delay tuning based on increasing repetition rates has already been described in several studies at the level of the cortex and auditory midbrain^24^,^42^,^43^,^44^,^45^,^46^,^47^,^48^. The main difference between our experiment and previous studies investigating the impact of stimulus repetition rate on neuronal specificity is in the nature of the sound sequences used for stimulation (i.e. natural (this study) vs. seminatural streams (previous studies)). One previous study compared neuronal responses elicited with call-echo elements with responses to a “semi-natural echolocation sequence”^47^. This approach rendered results that are comparable to ours, but the echolocation sequence that they used as stimulus was fundamentally different from the one of the present study. For example, their “semi-natural echolocation sequence” was composed of one natural call repeated with a constant call intensity and intercall time interval of 83.3 ms (12 Hz). However, spectrotemporal call parameters, intensities and intercall time intervals are not constant during an approach flight (Fig. 1). Despite differences in the stimulus settings, the study by Bartenstein^47^ reported a sharper delay tuning at the cortex of *Phyllostomus discolor*, an evolutionarily close relative of *C. perspicillata* when stimulating with high repetition rates. In comparison to their results, where only 50% of cortical units were more sharply tuned in response to the “semi-natural sequence”, all units (n = 149) of the present study were suppressed in the “natural situation”. Based on these differences, one could argue that suppression effects could be stronger when studied with natural (as opposed to seminatural) echolocation sequences. Supporting this idea, a recent electrophysiological study showed stronger selectivity to natural than to artificial echolocation streams in subcortical neurons of the big brown bat (*Eptesicus fuscus*)^49^. These results, together with ours show that the temporal context and spectrotemporal profile of the call-echo elements affect the response selectivity. Strong selectivity to natural sequences is already present at the level of the midbrain (specifically the superior colliculus). It remains elusive whether the sharper tuning reported here is the result of cortical suppression only or whether it profits from suppression effects that are inherited from subcortical structures. Note that suppression built in the cortex could also shape responses measured in subcortical structures, because of corticofugal projections^50^,^51^.

Regardless of its origin, our results show that cortical neurons can profit from forward suppression which is induced in response to natural echolocation sequences. Suppression acts as a physiological filter that operates in the time domain and that ensures sharp target-distance tuning and a more distinct topographic organization of echo delays. In addition, suppression increases the delay processing range that is covered by the neurons (Fig. 4). Therefore, suppression should be seen as a mechanistic tool rather than a limiting element in cortical processing. Bats are well suited animal models for investigating cortical suppression, because these animals usually encounter highly repetitive stimuli of specific behavioural relevance. In this regard, bats may provide the means to resolve the resolution-integration paradox stating that neurons can either integrate information over long time periods or maintain precise temporal resolution^52^.

## Methods

### Animals

Electrophysiological experiments were performed in ten adult bats (5 females and 5 males) of *Carollia perspicillata* bred in a colony of the Institute for Cell Biology and Neuroscience (Frankfurt University). The animal use complies with all current German laws on animal experimentation and it is in accordance with the Declaration of Helsinki. All experimental protocols were approved by the Regierungspräsidium Darmstadt (experimental permit # F104/57).

### Recording of echolocation signals and their preparation for neurophysiology stimulation

For recording natural echolocation sequences, the bat was fixated in a pendulum^10^. An ultrasound sensitive microphone (CM16/CMPA, Avisoft Bioacoustics, Germany) was medially positioned above the animal’s head. The microphone was adjusted as close as possible to the ears (~4 cm) to measure the calls and echoes as they would reach the ears of the animal. Its membrane was directed towards the forward swing trajectory. The microphone had a sensitivity of 50 mV/Pa and an input-referred self-noise level of 18 dB SPL. It was connected to a sound acquisition system (UltraSoundGate 116Hm mobile recording interface, +Recorder Software, Avisoft Bioacoustics, Germany) for sound digitalization at 375 kHz (16 bit precision). For offline analysis, digitalized signals were stored in a computer. The bat was swung (total distance = 4 m) towards a smooth, well reflecting acrylic glass wall (50 × 150 cm). During the swing, the animal broadcasted sequences of calls. A specific sequence representing a variable range of echo delays between 22.8 and 1.1 ms was chosen (Supplementary Table 1) to serve as acoustic stimulus for electrophysiological recordings.

The echolocation sequence was resampled from 375 kHz to 384 kHz and background noise was filtered via “Noise Reduction” (FFT length 256; precision 16) with the software Avisoft SAS Lab Pro (Avisoft Bioacoustics, Germany). The “noise reduction” function of Avisoft SAS Lab Pro filters the noise below a certain threshold in the frequency domain. The echolocation calls, together with its echoes, were above that threshold and therefore their spectro-temporal structure was not affected. Remaining artifacts from background noise were filtered with an elliptic filter (order 8) in the software BatSound (Pettersson Elektronik AB, Sweden). Different “stimulus situations” were prepared and played to the anaesthetized animal. In the “sequence situation” the natural echolocation sequence was presented. In the “element situation” individual pulse-echo elements of the natural echolocation sequence were presented without sequence context and a 400 ms interstimulus time interval. The natural echolocation sequence was segmented into its call-echo elements using a custom-written Matlab script (R2009b) and all call-echo elements as well as the natural echolocation sequence were saved as *wav* files. For investigating the relevance of the call-echo element order in the sequence we temporally reversed the natural echolocation sequence with the “reverse” function of BatSound (“reversed situation”). To ensure that each call was followed by an echo and for preserving the original spectrotemporal properties of the call-echo elements (i.e. that they were downward frequency modulated) successive call-echo elements were temporally reversed. The reversed natural echolocation sequence (“reversed situation”) as well as the natural echolocation sequence (“natural situation”) were randomly presented to the anaesthetized animal.

During electrophysiological recordings, acoustic stimuli were played at a sampling rate of 384 kHz with an Exasound E18 sound card (ExaSound Audio Design, Canada). Each *wav* file (containing either single call-echo elements or the entire sequence) was multiplied by a fading function in which energy increased/decreased smoothly over a time window of 0.5 ms. The latter ensured that no sound artifacts (clicks) were produced by the speaker when playing the natural files. Additionally, the output of the speaker was recorded when the sound files were played and the spectrogram and oscillograms were inspected to look for possible artifacts (with the software BatSound and Avisoft SAS Lab Pro). We could not observe any stimulation artifacts. The audio signals were transferred to an audio amplifier (Rotel power amplifier, RB-850). The bat was stimulated with a calibrated speaker (ScanSpeak Revelator R2904/7000, Avisoft Bioacoustics, Germany) located 15 cm from the bat’s ear. A speaker response curve used for calibration was measured with a ¼-inch Microphone (Brüel&Kjaer, model 4939, Denmark) which was connected to a custom-made microphone amplifier.

Frequency-level receptive fields, were measured using pure tone stimuli of 2 or 10 ms duration (0.5 ms rise-fall time). Used frequencies ranged from 5–95 kHz and the sound pressure levels were between 30–90 dB SPL. Sound levels were adjusted based on the speaker’s calibration curve. To be able to average the neuronal response, each frequency-level combination was presented 5 times in a randomized fashion and with a 400 ms interstimulus time interval.

Delay-tuning curves were measured with pairs of downward frequency modulations (FM) of 2 ms duration (0.5 ms rise-fall time). In the artificial FMs, the frequency fell from 99 kHz to 49.5 kHz and had its peak energy at 66 kHz. The FM mimicking the call was kept constant at 80 dB SPL and the FM mimicking the echo was adjusted from 60–80 dB SPL in 10 dB intervals. Echo delays between call and echo (from FM start to consecutive FM start) ranged between 2–22 ms. Each echo delay - echo sound pressure level combination was presented between 15–20 times in a randomized fashion and with a 400 ms interstimulus time interval. A delay tuning curve was also calculated using an example natural call that contained all the echolocation harmonics.

To record and to average the neuronal response each “stimulus situation” (“element”, “sequence”, “natural” and “reversed”) was played randomly 15–20 times at intervals of 400 ms. Three different sound levels were used while presenting the sequence or its elements. The loudest sequence contained elements whose levels ranged between 36–77 dB SPL (Supplementary Table 1). This sequence was attenuated by 10 dB or 20 dB to produce fainter stimulation streams.

### Data acquisition and analysis

Electrophysiological recordings took place in a sound-proofed and electrically-shielded chamber. Neuronal responses were recorded in the left and right hemispheres of the bats. For anaesthesia, bats were subcutaneously injected with a mixture of ketamine (10 mg/kg Ketavet, Pharmacia GmbH, Germany) and xylazine (38 mg/kg Rompun, Bayer Vital GmbH, Germany). A longitudinal midline incision was made through the skin overlying the skull. Muscle tissue, covering dorsal and temporal parts of the skull, was removed. A craniotomy above the cortical region corresponding to the high frequency area^53^ gave access to auditory neurons. For fixation of the bat’s head, a custom-made metal rod (1 cm length, 0.1 cm diameter) was glued onto the skull using dental cement (Paladur, Heraeus Kulzer GmbH, Germany). Each animal was used for chronical recording sessions that lasted up to 8 h over a period of several days.

For electrophysiology, two electrode types were used. (i) commercially available tungsten microwire arrays with 16 electrodes organized in 2 × 8 (Tucker Davis Technologies, USA). The arrays had an electrode spacing of 250 μm and a row spacing of 500 μm. Before penetration, the *dura mater* was carefully removed. Because of the array size, positioning the electrodes initially pushed the cortex downwards, therefore the recordings began only after the cortex had recovered its position (i.e. after 20–30 min, when all the electrodes were already inside the cortex). (ii) custom-built glass electrode arrays of up to 6 channels organized in a row. Glass electrodes (resistance 1–10 MΩ when filled with 3 Mol KCl) were pulled from borosilicate capillaries (GB120F-10, Science Products, Germany) with a Flaming/Brown horizontal puller (P97, Sutter, USA) and they were glued together in a fanshape pattern, ensuring an electrode tip spacing of 250 μm. Neuronal data acquisition used a wireless multichannel recording system (Multi Channel Systems MCS GmbH, Germany) at a sampling rate of 20 kHz per channel and 16 bit precision. Initially we recorded with the microwire arrays but we switched later to the custom-built glass electrode arrays because of several reasons. First, the resistance of the glass electrodes was much higher giving us higher recording quality. Second, recording with glass electrodes was less invasive for the animals because we did not have to remove the dura mater, and therefore each animal could be used for several days. Neuronal responses were analysed in 149 multi-units. 38 units were recorded with the microwire array and 111 units with the glass electrode array. Neuronal tuning was comparable in both recording approaches. For detecting spike events we took a multi-unit specific threshold based on the spike amplitude and used this threshold throughout the recordings for that particular multi-unit. Due to the fact that we used the same threshold within one multi-unit and throughout the stimulation protocol we can confirm that we picked up the same multi-unit activity for each stimulus.

To investigate the impact of the interstimulus time interval of the sequence we compared the neural response to the “sequence situation” and to the “element situation”. For achieving this, we realigned the spike-times elicited in response to the individual call-echo elements based on the position of the corresponding call-echo element in the echolocation sequence. For example, the response to the first call-echo element was temporally positioned in the front followed by the spikes elicited by the second call-echo element etc… The result of this realigning procedure was an “expected neural response” to the natural sequence that is based on the responses to the single call-echo elements. For the post-stimulus time histograms (PSTHs) used to process the sequence stimulation data the binsize was set 5 ms. Sign tests were calculated in Matlab (2014) and the remaining statistics in GraphPad Prism 5 (GraphPad Software, USA; *p < 0.05; **p < 0.01; ***p < 0.001). The time window for median response calculation ranged from 150 ms after beginning of the sequence to the end of the sequence (1550 ms). The first 150 ms were not considered, to avoid frequently occurring strong initial responses which we considered not to be related to delay tuning.

## Additional Information

**How to cite this article**: Beetz, M. J. *et al*. Temporal tuning in the bat auditory cortex is sharper when studied with natural echolocation sequences. *Sci. Rep.* **6**, 29102; doi: 10.1038/srep29102 (2016).

## Supplementary Material

Supplementary Information
