# Supplementary material for: Temporal tuning in the bat auditory cortex is sharper when studied with natural echolocation sequences
Source: Sci Rep. 2016 Jun 30;6:29102. doi: 10.1038/srep29102 (PMC4928181; doi:10.1038/srep29102)
Supplement: Supplementary Information [file srep29102-s1.pdf]

***Title*: Temporal tuning in the bat auditory cortex is sharper when studied with natural echolocation sequences**

***Authors*:** M. Jerome Beetz (*) [1], Julio C. Hechavarría [1], Manfred Kössl [1].

***Affiliations*:** [1:]Institut für Zellbiologie und Neurowissenschaft, Goethe-Universität, 60438, Frankfurt/M., Germany.

* Corresponding author

***Contact*:**

M. Jerome Beetz

Email: Jeromebeetz@arcor.de

Institut für Zellbiologie und Neurowissenschaft, Max-von-Laue-Straße 13, 60438, Frankfurt/Main, Germany, Tel.: +49 69798 42066

Supplementary Table 1 Sequence paramters (refers to Fig. 1)
Calculations were done with Avisoft SASLab Pro (Avisoft Bioacoustics, Germany).
BW = bandwidth; dur = duration; duty cycle = call duration/call interval; f = frequency; int = intensity; rms = root mean square

# call	dur	pulse interval	delay	int pulse	int echo	duty cycle	peak f	min f	max f	BW
	[ms]	[ms]	[ms]	rms [dB SPL]	rms [dB SPL]	[%]	[kHz]	[kHz]	[kHz]	[kHz]
1	0.87		22.8	63	38	2.94	76	65.6	89.1	23.4
2	1.073	29.62	22.7	72	36	2.46	77.6	59.6	94.9	35.3
3	0.839	43.573	22.2	69	36	2.94	77.2	62.4	94.5	32
4	0.813	28.573	22	68	36	1.53	71.6	59.6	94.6	35
5	0.766	53.099	21.8	72	36	2.52	76.3	64	92.4	28.3
6	0.896	30.422	21.4	73	40	1.72	73.9	58.9	95.8	36.8
7	0.906	51.964	20.8	73	36	2.83	77.5	65.9	91.7	25.7
8	1.109	31.974	20.1	75	38	2.03	69.4	59.8	91.2	31.3
9	0.901	54.594	19.2	72	38	2.33	77.2	60.1	92.6	32.4
10	0.885	38.688	18.4	77	49	1.55	60.6	55.3	83.5	28.2
11	0.932	57.083	17	74	47	2.50	70.7	56.9	86.9	29.9
12	0.807	37.286	15.9	71	49	1.20	56.7	52.6	80.8	28.2
13	0.698	67.036	14	73	48	0.92	60.1	54.6	80.7	26.1
14	0.849	75.818	11.6	72	57	1.14	57.1	53.8	74.5	20.6
15	0.948	74.385	9.4	73	58	1.19	59.8	54.6	85.5	30.9
16	1.047	79.75	7.2	77	60	2.29	72.5	60	94.4	34.4
17	0.792	45.802	6	69	56	1.55	77.4	63.3	93.8	30.5
18	0.677	50.974	5	65	53	2.01	82.9	62.3	95.8	33.5
19	0.745	33.677	4.3	66	56	1.31	86.9	72.7	94	21.2
20	0.703	57.016	3.4	65	50	2.09	89.9	77.3	94.5	17.1
21	0.687	33.698	3	63	56	1.85	89.8	79.7	94.5	14.8
22	0.823	37.146	2.5	71	63	1.99	90.6	79.1	94.3	15.2
23	0.677	41.266	2	66	66	2.53	90.8	79.9	94.4	14.5
24	0.63	26.708	1.8	65	70	2.21	91.2	76.3	94.2	17.8
25	0.495	28.516	1.6	62	67	1.12	83.5	64.9	98.5	33.6
26	0.516	44.062	1.5	63	63	1.58	82.8	64.6	92.5	27.9
27	0.62	32.667	1.3	68	68	1.79	83.5	76.4	94.3	17.8
28	0.552	34.547	1.2	66	73	1.31	90.8	71.1	94.2	23
29	0.651	41.995	1.1	69	76	2.31	84	65.8	92.9	27.1
30	0.667	28.125	1.1	74	76	1.95	83.2	57.9	100.4	42.5
31	0.651	34.141	1.1	74	77		67.9	57.3	93.6	36.2

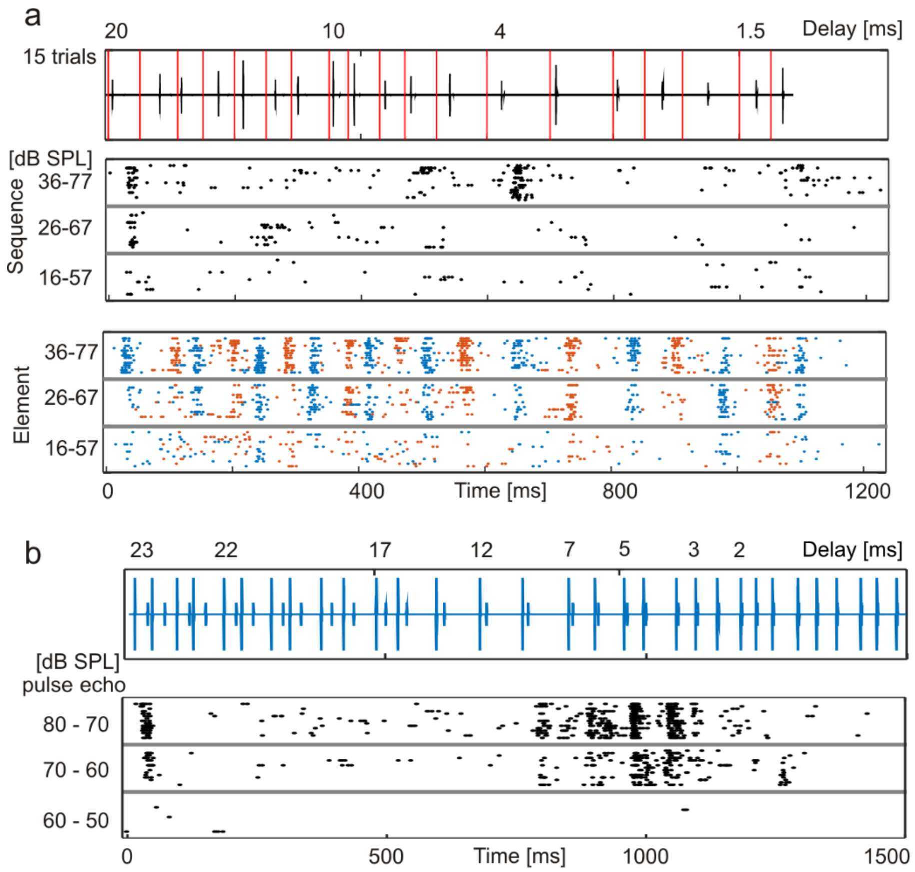

Supplementary Fig.1 Cortical suppression occurring to animal specific echolocation sequence and delay tuning to seminatural echolocation sequence (refers to Fig. 2).

(a) Neuronal response of the multi-unit from figure 2 when stimulated with a “natural sequence” obtained from the animal that was electrophysiologically recorded. Note that the strongest response in the “sequence situation” occurs at 4 ms delay which fits to the tuning properties shown in figure 2b and 2c. Organization of the figure as in figure 2c. **(b)** Neuronal response of the multi-unit to a seminatural echolocation sequence. The sequence was constructed with a natural echolocation call. The call was attenuated by 10 dB and used as an echo. Call and echo positions were adjusted to the positions from the “natural situation” in figure 2. Thus the temporal properties (intercall time intervals and echo delays) were equal to the “natural situation” in figure 2. The intensities of the calls and echoes were consistent throughout the sequence. When comparing the response pattern of **(b)** with that of figure 2c then it becomes clear that the overall response pattern was comparable although the multi-unit responded with more spikes in the “seminatural situation”. Intensity differences together with spectral differences of the calls could be responsible for the slightly stronger response in the “seminatural situation”.

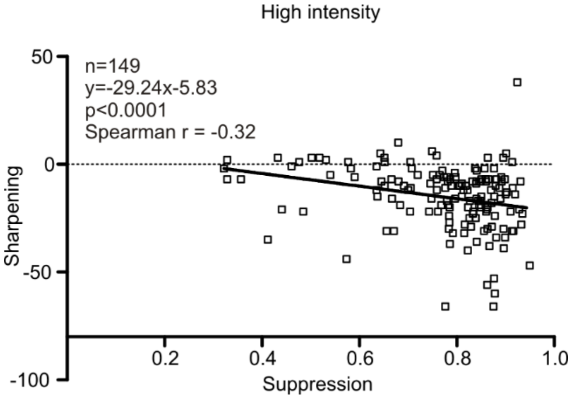

Supplementary Fig. 2 Linear correlation between the suppression rate and the bandwidth difference at the highest intensity level (refers to Fig. 3). * = p < 0.05; ** = p < 0.01; *** = p < 0.001

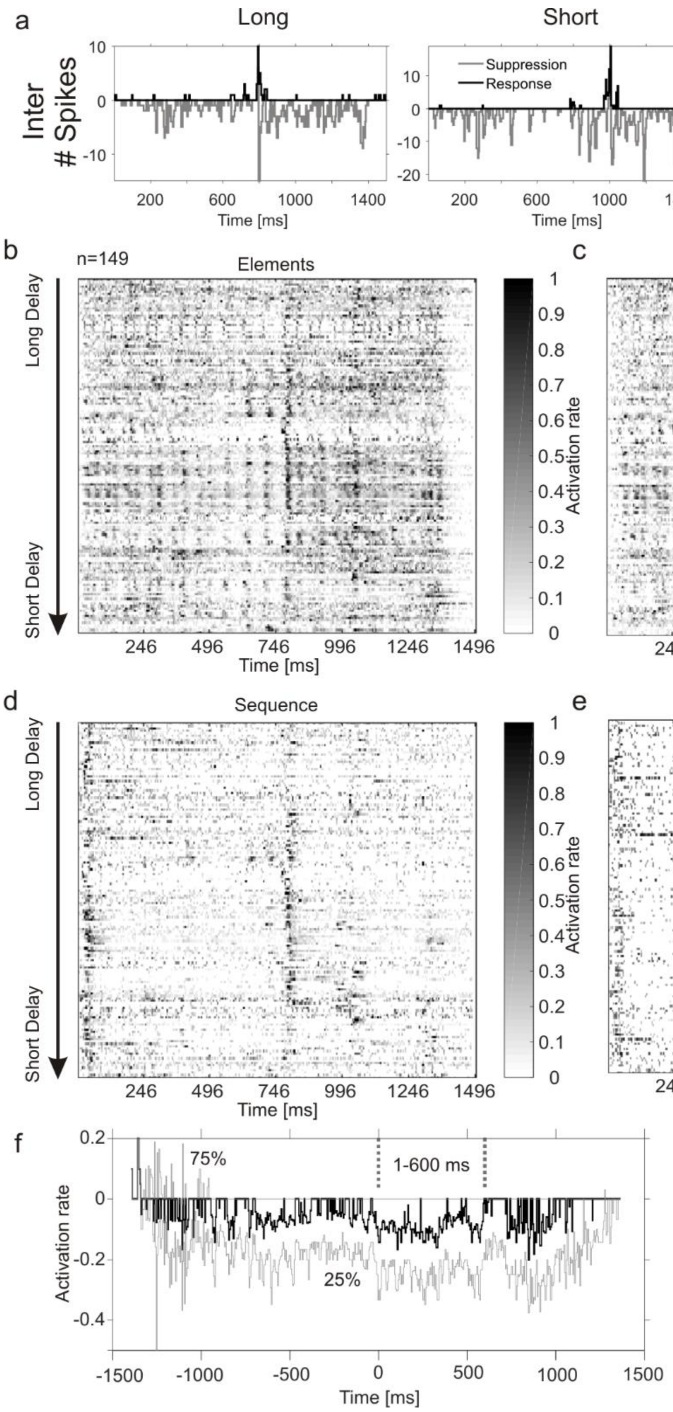

Supplementary Fig. 3 Forward suppression predominantly occurs right after the best response also at intermediate intensity level (refers to Fig. 5)

(a) Intermediate intensity level PSTH of two example units in response to the natural echolocation sequence (black) and a suppression PSTH (grey). Note that high suppression often occurs close to the strongest response.

(b) Color map of normalized PSTHs from each unit (organized with decreasing best delays along the y-axis) in response to the element situation.

(c) Color map of normalized suppression PSTHs from each unit.

(d) Color map of normalized PSTHs from each unit in response to the sequence situation. Note the extremely sharp activity areas (dark spots).

(e) Color map of normalized facilitation PSTHs from each unit. Note the facilitation pattern resembles the activation pattern in the sequence situation.

(f) Median contrast PSTH calculated from temporally aligned contrast PSTHs of each untit. Alignment occurred, thus best response in the sequence situation correspond to time point 0. Suppressive effects are weaker than in the high intensity level (compare Fig. 5g). One prominent suppressive area at 1-600 ms after the best response occurs. In comparison to figure 5g no peak appeared at time point 0, because of decreased

overall activity of the units at that intensity level. Therefore, the median calculation filtered tiny responses out of the median contrast PSTH.

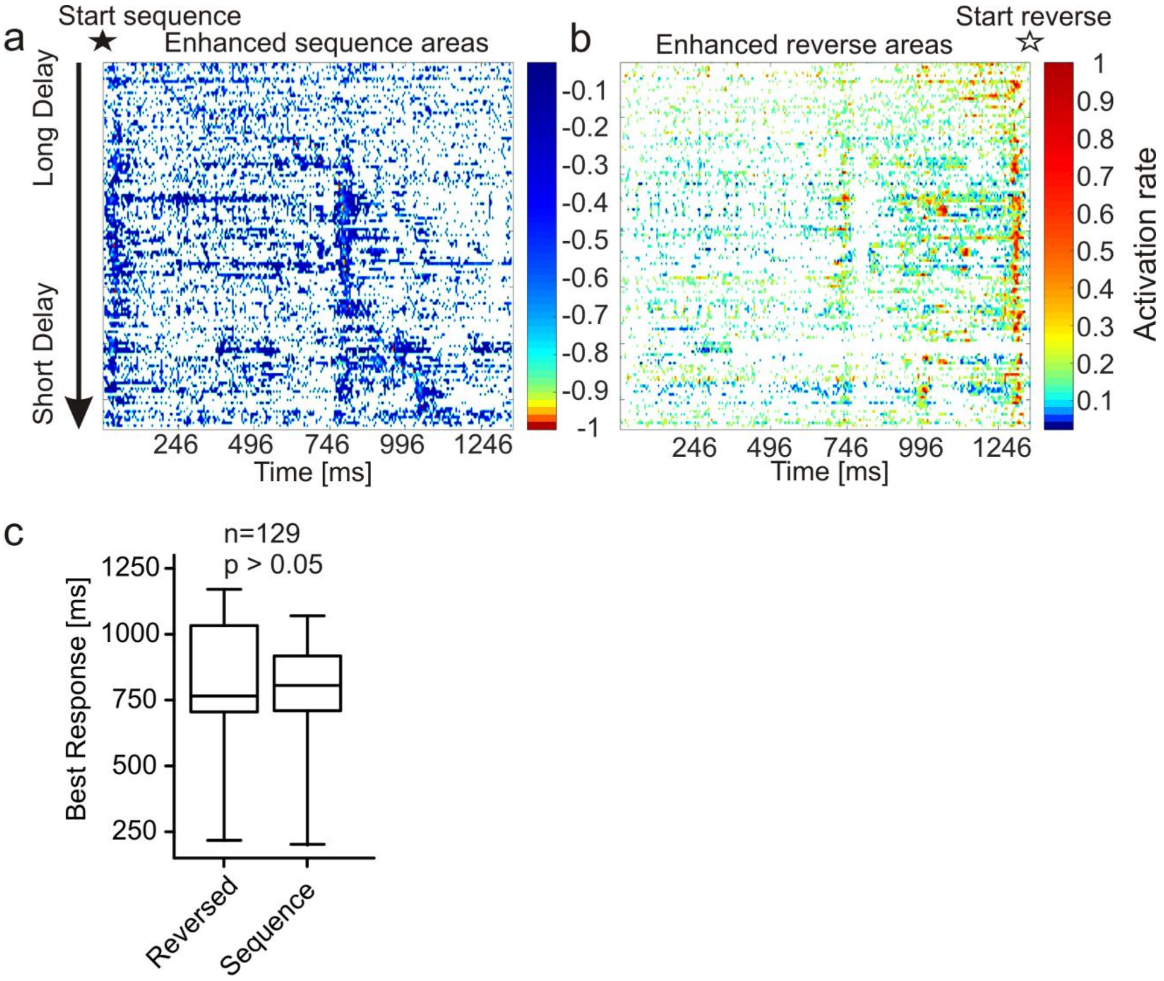

Supplementary Fig. 4 Forward suppression is the prominent suppression form (refers to Fig. 6)
(a, b) Color maps showing exclusively enhanced sequence areas **(a)** and enhanced reverse areas **(b)** bins.
(c) No significant best response shift occurred between sequence and reversed stimulation (Wilcoxon signed rank test: $p = 0.07$)
